# High-fidelity and clean nanotransfer lithography using structure-embedded and electrostatic-adhesive carriers

**DOI:** 10.1038/s41378-022-00476-x

**Published:** 2023-01-09

**Authors:** Zhuofei Gan, Jingxuan Cai, Zhao Sun, Liyang Chen, Chuying Sun, Junyi Yu, Zeyu Liang, Siyi Min, Fei Han, Yu Liu, Xing Cheng, Shuhui Yu, Dehu Cui, Wen-Di Li

**Affiliations:** 1 Department of Mechanical Engineering, University of Hong Kong, Hong Kong, China; 2 School of Microelectronics, Southern University of Science and Technology, Shenzhen, China; 3 School of Biomedical Engineering, Sun Yat-sen University, Guangzhou, China; 4 The Shenzhen Institute of Advanced Electronic Materials, Shenzhen Institutes of Advanced Technology, Chinese Academy of Sciences, Shenzhen, China; 5 Department of Materials Science and Engineering, Southern University of Science and Technology, Shenzhen, China

**Keywords:** Nanostructures, Organic-inorganic nanostructures

## Abstract

Metallic nanostructures are becoming increasingly important for both fundamental research and practical devices. Many emerging applications employing metallic nanostructures often involve unconventional substrates that are flexible or nonplanar, making direct lithographic fabrication very difficult. An alternative approach is to transfer prefabricated structures from a conventional substrate; however, it is still challenging to maintain high fidelity and a high yield in the transfer process. In this paper, we propose a high-fidelity, clean nanotransfer lithography method that addresses the above challenges by employing a polyvinyl acetate (PVA) film as the transferring carrier and promoting electrostatic adhesion through triboelectric charging. The PVA film embeds the transferred metallic nanostructures and maintains their spacing with a remarkably low variation of <1%. When separating the PVA film from the donor substrate, electrostatic charges are generated due to triboelectric charging and facilitate adhesion to the receiver substrate, resulting in a high large-area transfer yield of up to 99.93%. We successfully transferred the metallic structures of a variety of materials (Au, Cu, Pd, etc.) with different geometries with a <50-nm spacing, high aspect ratio (>2), and complex 3D structures. Moreover, the thin and flexible carrier film enables transfer on highly curved surfaces, such as a single-mode optical fiber with a curvature radius of 62.5 μm. With this strategy, we demonstrate the transfer of metallic nanostructures for a compact spectrometer with Cu nanogratings transferred on a convex lens and for surface-enhanced Raman spectroscopy (SERS) characterization on graphene with reliable responsiveness.

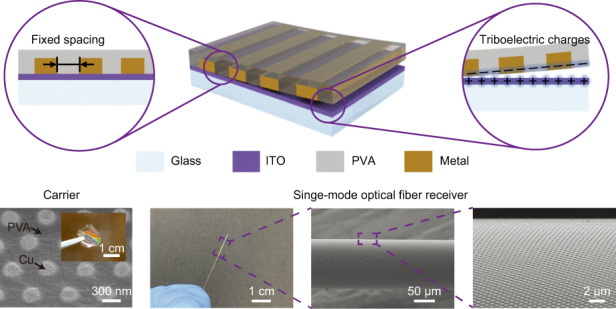

## Introduction

Nanoscale metallic structures and their potential applications are currently being investigated in many emerging fields, such as the fields of plasmonics^1–3^, electronics^4,5^, and biosensors^6,7^. However, a variety of these applications require the patterning of nanostructures on unconventional substrates, such as curved optical components (optical fiber, lens, etc.) and soft biomimetic materials, for which traditional nanofabrication strategies, such as photolithography, plasma etching, and vacuum deposition, are difficult to apply. For example, spin-coating photoresist onto curved surfaces in a uniform layer is challenging, and high-vacuum evaporation is not suitable for porous substrates. Therefore, patterning metallic nanostructures with high fidelity and quality on unconventional surfaces still represents a substantial challenge. Recently, nanotransfer lithography (NTL) has become one of the most adopted methods for patterning on unconventional surfaces due to its process simplicity, high throughput, and cost-effectiveness^8–14^.

Generally, the transfer carrier plays an essential role in the transfer process due to its close relationship with both the donor and receiver substrates. In most published nanotransfer research, functional materials were first deposited on nanostructured carriers by evaporation or sputtering and then transfer-printed onto specific receivers^15^. However, this approach normally introduced an undesirable edge roughness on the transferred pattern because the material was deposited on the surfaces of nanostructures with no confinement. It also limits the transformed pattern geometries to planar structures, making thick and three-dimensional (3D) structures difficult to transfer. In addition, chemical adhesive promotors, which can form chemical bonds via surface treatments to increase adhesion, are also needed to improve the transfer yield^16,17^. However, most chemical adhesive media are environmentally harmful, and their residues may negatively affect the performance of fabricated devices. Although adhesive-layer-free nanotransfer is also feasible, the corresponding receivers are generally textiles^18^, thermoplastic polymers^19^, or sticky scotch tape^20^. To date, it remains a challenge to develop an NTL process that simultaneously satisfies three conditions: (1) high fidelity and high transfer yield, (2) no additional residues, and (3) arbitrary receivers.

Herein, we develop a high-fidelity, clean, structure-embedded, and electrostatic-adhesive NTL (SENTL) process to address the above challenges. As the transfer carrier, a water-soluble polyvinyl acetate (PVA) film embeds the transferred structures and maintains their original order to ensure high fidelity with <1% spacing variation. The PVA transfer carrier also provides triboelectric charges generated from the peeling off process, facilitating adhesion to the receiver substrate, which leads to a high transfer yield of 99.93%. The encapsulating carrier is not only suitable for planar structures of different materials (Au, Cu, Pd, etc.) but also enables the transfer of high-aspect-ratio (>2) gratings with 600-nm thickness and 3D structures. Moreover, the electrostatic-adhesive approach leaves no residues after dissolving the PVA carrier. In addition, the flexible carrier film enables full contact with a variety of receivers, including rigid, soft, planar, and curved surfaces. As a demonstration, a large-area nanoscale pattern is successfully transferred onto a single-mode optical fiber with a small curvature radius of 62.5 μm. We also demonstrate the transfer of nanogratings onto a convex lens as a light-dispersive element for a compact spectrometer and gold nanodisks onto graphene for surface-enhanced Raman spectroscopy (SERS) analysis.

## Results

### Fabrication process of the high-fidelity and clean SENTL

**Fig. 1:**
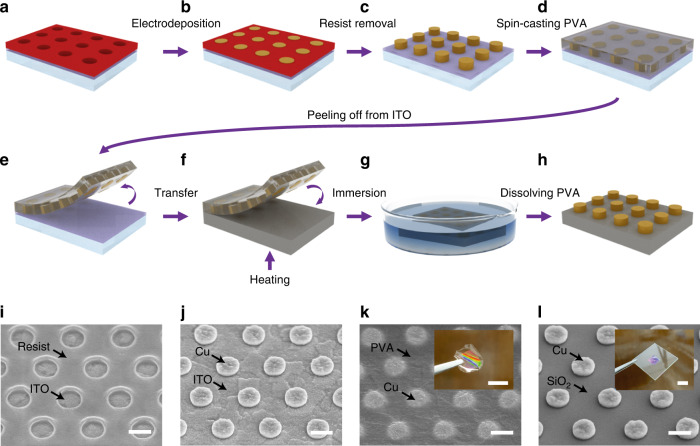
Process flow of SENTL applied to electrodeposited metallic nanostructures. **a** Patterning in the resist on ITO substrate, **b** electrodeposition within the resist pattern, **c**, **d** formation of the structure-embedded film by spin-casting PVA aqueous solution after resist removal, **e** peeling-off of the PVA carrier with the embedded nanostructures, **f** attachment of the PVA carrier onto receiver substrates, **g**, **h** nanostructures transferred after dissolving PVA in water. SEM images of **i** imprinted nanoholes in the resist on ITO, **j** electrodeposited Cu nanodisks on ITO, **k** Cu nanodisk-embedded PVA film, and **l** transferred Cu nanodisks on a glass substrate. Scale bars, 300 nm (**i**–**l**), 1 cm (inset **k**, **l**)

Using electrodeposited metallic nanostructures as an example, the process flow of SENTL is schematically illustrated in Fig. 1a–h. First, a nanoscale structure is patterned in the resist on indium tin oxide (ITO) glass by nanoimprinting or interference lithography, and electrodeposition on the exposed ITO surface produces metallic nanostructures^21,22^. Then, after removing the resist, PVA aqueous solution is spin-cast on the patterned substrate and cured to form a layer of PVA film that encapsulates the nanostructures. Next, the PVA film with embedded nanostructures can be easily peeled off from the ITO substrate while effectively maintaining the order of the nanostructures. During this process, triboelectric charges are also induced by the detachment of the PVA film from the ITO surface. Then, the PVA film is attached to the receiver substrate, and the induced charges contribute to electrostatically enhanced binding between the film and the receiver surface, resulting in a high transfer yield. Finally, after dissolving PVA in water, the metallic nanostructures are firmly transferred onto the receiver.

Scanning electron microscopy (SEM) is implemented to characterize the pattern morphologies at different fabrication steps. Figures 1i, j show the imprinted nanoholes in the resist and electrodeposited Cu nanodisks on the ITO substrate, respectively, featuring complementary morphologies due to the resist confinement. Figure 1k shows the separated PVA film with embedded Cu nanodisks. The film has a thickness of 15 μm, as observed in Fig. S1. Figure 1l shows the transferred Cu nanodisks on a glass substrate after dissolving PVA. The inset photographs in Fig. 1k, l display the complete pattern with light diffraction.

### High-fidelity order maintenance by polymeric embedding and high transfer yield owing to electrostatically enhanced adhesion

**Fig. 2:**
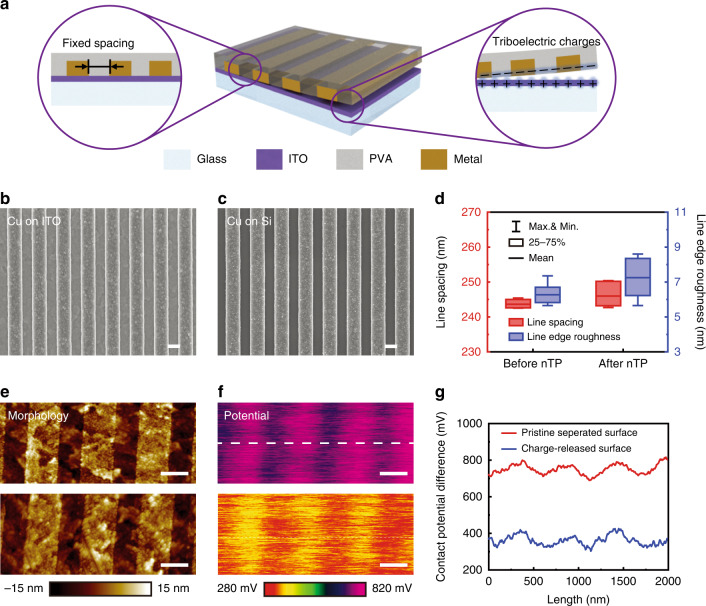
**a** Schematic illustration of the high-fidelity lateral dimension maintenance and high transfer yield due to triboelectric electrostatic adhesion. **b**, **c** SEM images of Cu nanogratings before and after SENTL transfer, respectively. **d** Statistical box plot with the mean, maximum, minimum, and 25–75% range of the distribution of the line spacing and line edge roughness of Cu nanogratings before and after transfer. **e**, **f** KPFM surface topography and surface potential mapping of the PVA carrier film with embedded Au nanogratings immediately after separation and after charge release, respectively. **g** KPFM surface potential difference variation before and after charge release. Scale bars: 300 nm

In the SENTL process, the PVA carrier plays a crucial role in maintaining the nanostructure order and providing reliable adhesion to the receiver substrate (Fig. 2a). The cured PVA exhibits moderate elasticity under small stretching that occurs during peeling-off and sample handling, which can help to maintain the nanostructure dimensions and the horizontal spacing between neighboring nanostructures in the short range to achieve high transfer fidelity. Moreover, its flexibility can allow the necessary deformation for conforming to nonplanar or irregular receiver surfaces.

When the aqueous PVA solution is spin-cast on prefabricated nanostructures on the donor substrate, it flows and fills the gap among nanostructures. Then, the cured PVA encapsulates nanostructures into a solid film, maintaining the order. In the subsequent peeling, transferring and attaching steps, the solid PVA well-maintains the local spacing by elastically responding to temporary mechanical stress. We perform the SENTL process to transfer 600-nm-period Cu gratings from the ITO donor to the Si receiver (Fig. 2b, c) and quantitatively characterize the spacing variation before and after the transfer using SEM. A box chart plotted in Fig. 2d shows that the average line spacing between gratings changes from 243.6 nm to 245.9 nm. The spacing change is <1%, indicating that SENTL possesses a high fidelity. The 3*σ* deviation of the line edge roughness (3*σ*-LER) also exhibits a small change of less than 1 nm from 6.3 nm to 7.2 nm. The slight increase in line spacing and LER may be caused by the local deformation of the edges of the metallic structures, which are vulnerable to external forces during the peel-off separation and PVA removal process.

On the other hand, triboelectric charges induced on the surface of the PVA carrier during its separation from the donor ITO substrate contribute to significantly improving the transfer reliability with enhanced electrostatic-adhesive force. According to the electron transfer mechanism and triboelectric series^23–25^, electrons transfer from the ITO to the PVA carrier at the interface when the two are in contact, as the ITO is prone to donating electrons and the PVA prefers to accept them. When the PVA film is peeled off from the ITO substrate, the pristine PVA surface surrounding the transferred nanostructure is exposed and becomes negatively charged with the total charge amount proportional to the exposed PVA surface area^26,27^.

To confirm the existence of the induced triboelectric charges, the surface potential variation is quantitatively characterized using Kelvin probe force microscopy (KPFM). We measure the morphologies and contact potential differences on the PVA carrier film immediately after separation and after releasing the surface charges (details in Methods), as shown in Fig. 2e, f, respectively. Although both exhibit similar surface morphologies, on the pristine PVA surface, the surface potential is approximately up to 750 mV, while that on the charge-released surface is ~300 mV, indicating that more negative charges exist on the pristine PVA surface (Fig. 2g). The charges on the PVA carrier enable electrostatic adhesion enhancement when attached to the receiver substrate due to dielectric polarization. As a result, centimeter-scale nanopatterns can be completely transferred by SENTL with a transfer yield up to 99.93%, as examined at 5 different positions of the 1-cm^2^-area transferred nanodisk pattern (Fig. S2).

### Structural and material versatility in the SENTL process

**Fig. 3:**
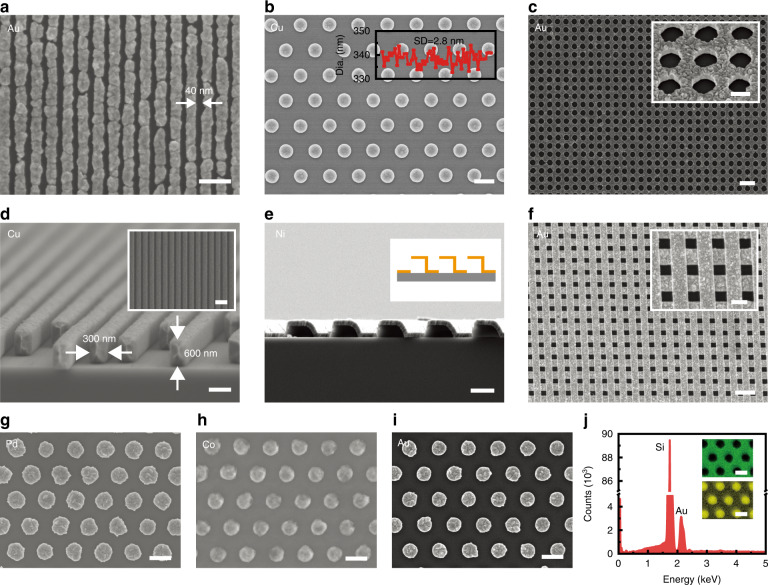
SEM images of various nanostructures transferred by SENTL. **a** High-resolution Au nanogratings with 40-nm spacing. **b** 700-nm-period Cu nanodisks of uniform diameters with a standard deviation of 2.8 nm. **c** 550-nm-period Au nanohole array. **d** High-aspect-ratio Cu nanogratings with 300-nm width and 600-nm height. **e** Quasi-3D Z-shaped Ni nanostructures. **f** Stacked-mesh nanostructure fabricated by double SENTL transfer. **g**–**i** Transferred 700-nm-period Pd, Co, and Au nanodisks, and **j** corresponding EDX spectrum in **i** indicating a residue-free transfer. Scale bars: 300 nm (**a**, insets in **c**, **f**), 500 nm (**b**, **d**, **e**, **g**–**j**), and 1000 nm (**c**, **f**, inset in **d**)

SENTL enables the transfer of nanostructures with various geometries and materials. Figure 3a shows the SEM image of transferred Au nanogratings on a Si substrate down to 40-nm spacing, indicating the high resolution that SENTL can achieve. SENTL also exhibits high fidelity in transferring discrete 2D nanopatterns. For example, an array of Cu nanodisks with hexagonal order is transferred onto a glass substrate, maintaining a pristine order and a uniform disk diameter with a standard deviation of 2.8 nm (Fig. 3b). Figure 3c presents a transferred continuous Au nanohole array.

By encapsulating the nanostructure into the transfer carrier, SENTL provides further opportunities to transfer challenging complex patterns, such as high-aspect-ratio and complex 3D nanostructures. Figure 3d shows the cross-sectional SEM images of 300-nm-width and 600-nm-height rectangular nanogratings on Si, featuring an aspect ratio of 2. Compared to most reported methods for transferring evaporated material with nanostructured carriers, SENTL is advantageous for transferring more than 100-nm-thick nanostructures on the subwavelength scale. In addition, using the transferred thick metallic pattern as an etching mask instead of using conventional metal evaporation and lift-off processes, the underlying substrates can be directly etched with a high aspect ratio. For example, using the transferred pattern (Figs. 2c and 3g) as the etching mask, the silicon substrate can be etched up to 800 and 1500 nm, respectively, as demonstrated in Fig. S3. Moreover, SENTL also supports the transfer of 3D nanostructures, such as Z-shaped gratings (Fig. 3e), as the PVA aqueous solution can fill into the complex gap and support the hollow structure after curing (Fig. S4). In addition, multistep layer-by-layer transfer is also applied in SENTL for fabricating stacked 3D nanostructures. As demonstrated in Fig. 3f, a stacked nanomesh can be constructed by orthogonally transferring the nanogratings twice, which has been reported in applications such as gas sensing due to its high specific area^28^. Using the stacked nanomesh as an etching mask, the square nanohole array pattern can also be transferred to the underlying silicon substrate, as shown in Fig. S5. No defects were observed on the etched pattern, indicating that the SENTL process left no residues.

Our SENTL process is compatible with the nanostructures of a wide range of materials. When applied to electrodeposited nanostructures, the SENTL process is demonstrated on nanostructures made of Au (Fig. 3a, c, i), Cu (Fig. 3b, d), Ni (Fig. 3e), and other metals that can be electrodeposited, such as Pd and Co (Fig. 3g, h). Energy-dispersive X-ray (EDX) spectroscopy results obtained from the transferred metallic structures further show that no polymer residue remains (Figs. 3j and S6) since the adhesive promoter in SENTL is the triboelectric charges and the transfer media is the water-soluble PVA, which contribute to the clean transfer.

### Receiver substrate compatibility in the SENTL process

**Fig. 4:**
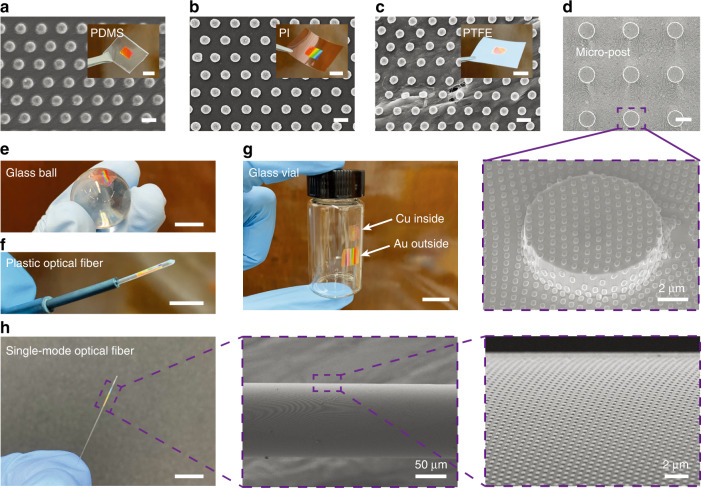
SEM images and photographs of SENTL-transferred nanostructures on various receiver substrates, including a **a** PDMS pad, **b** PI film, **c** PTFE film, **d** 10-μm diameter and 2-μm height Si micropost, **e** glass ball, **f** plastic optical fiber, **g** inner and outer wall of a glass vial, and **h** 125-μm diameter single-mode optical fiber. Scale bars: 500 nm (**a**–**c**), 10 μm (**d**), and 1 cm (**e**–**h**, insets in **a**–**c**)

SENTL can be applied on various receiver substrates, rigid or flexible, planar or curved, adhesive or nonadhesive, owing to its flexible PVA carrier and electrostatic adhesion. In addition to the successful transfer onto the rigid Si and glass substrates used in previous demonstrations, we also successfully transfer Cu nanodisks onto the polydimethylsiloxane (PDMS) soft pad and polyimide (PI) thin film (Figs. 4a, b), which could have potential applications in flexible wearable electronics^29–31^. In addition, SENTL also enables transfer on polytetrafluoroethylene (PTFE) substrates with an ultralow friction coefficient, as shown in Fig. 4c, demonstrating the transferred nanodisks firmly adhering to the rugged low-surface-energy surface and the maintained well-ordered arrangement. In addition, SENTL is also capable of fabricating hierarchical structures for security identification^32^ and superhydrophobic surfaces. As a demonstration, Fig. 4d shows the Cu nanodisks transferred onto a 10-μm-diameter and 2-μm-height micropost array.

Moreover, SENTL also provides opportunities to transfer nanostructures onto highly curved substrates, such as a 2-cm-diameter glass ball (Fig. 4e) and a 1-mm-diameter plastic optical fiber (Fig. 4f). Cu and Au nanodisk arrays can be transferred by SENTL onto both the inner and outer surfaces of a glass vial (Fig. 4g), which may have applications in directly detecting low-concentration molecules dispersed in a solution^33^. In particular, we demonstrate that SENTL can be used to transfer 700-nm-period and 350-nm-diameter Cu nanodisk arrays onto quartz optical fibers, producing a highly miniaturized optoelectronic platform^34–37^, for which conventional nanofabrication techniques face great challenges. As shown in Fig. 4h, a 125-μm-diameter single-mode optical fiber is wrapped with transferred nanodisks with high fidelity and a high transfer yield. It is worth noting that the SENTL process cannot be performed on substrates vulnerable to water since PVA removal requires water immersion or conductive substrates that easily release electrostatic charges, such as stainless steel (details in Methods section).

### Compact spectrometer with convex nanogratings and SERS analysis on graphene enabled by SENTL

**Fig. 5:**
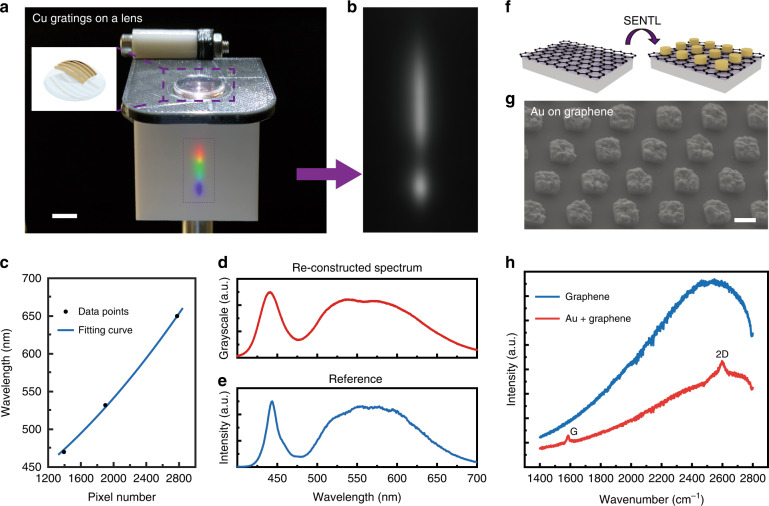
Compact spectrometer and SERS characterization enabled by SENTL. **a** Photograph of rainbow-like stripe image formed by dispersing and focusing the light from a white LED using the transferred nanogratings on a convex lens. **b** Grayscale image of the stripe captured by a monochromatic CMOS detector. **c** Plot that maps the pixel position to the calibrated wavelength. **d** Reconstructed spectrum obtained from the grayscale image. **e** Reference spectrum measured by a commercial spectrometer. **f** Schematic diagram and **g** SEM image of transferred Au nanodisks on monolayer graphene. **h** Raman spectra showing an enhancement in the Raman signatures of Au-covered graphene compared to that of bare graphene under 785-nm excitation. Scale bars: 300 nm (**g**), and 1 cm (**a**)

SENTL enables the fabrication of unconventional devices that feature functional nanostructures on nonplanar, flexible, or irregular substrates. We first demonstrate a compact spectrometer that is based on a convex lens with the surface patterned with diffractive gratings so that wavelength dispersion and imaging can be performed using a single component, as shown in Fig. 5a. SENTL can be used to transfer 600-nm-period Cu nanogratings onto the convex lens. The light from a white LED is dispersed by the grating and focused by the lens, forming a rainbow-like stripe image on the screen. By recording this image using a monochromatic complementary metal oxide semiconductor (CMOS) detector (Fig. 5b) and mapping the spatial position to the corresponding wavelength (Fig. 5c), we can reconstruct a spectrum of the incident light beam (Fig. 5d). The relationship between the spatial position and the corresponding wavelength is determined through calibration using narrow-band light beams of known wavelengths^38^ (e.g., 470, 532, and 650 nm, Fig. S7). The reconstructed spectrum in Fig. 5d exhibits good consistency with the spectrum obtained using a commercial spectrometer (Fig. 5e).

We also transfer Au nanodisks, which can greatly enhance Raman spectroscopic signals through local electrical field concentration, onto atomically thin materials for SERS characterization (Fig. 5f). Figure 5g shows Au nanodisks transferred onto monolayer graphene supported by a SiO_2_-coated substrate. As demonstrated in Fig. 5h, the transferred Au nanodisks significantly enhance the weak Raman signature on bare graphene and quench the fluorescence of the SiO_2_ substrate.

## Discussion

In summary, we report a facile and efficient nanotransfer lithography approach for achieving clean, high-fidelity and high-transfer-yield nanopatterning on diverse substrates with no polymeric residues. As the water-soluble transfer carrier, the spin-casted PVA film effectively embeds the prefabricated nanostructures and provides electrostatic adhesion for transfer printing. With the SENTL strategy, we can achieve a high fidelity with <1% transfer spacing variation and a high transfer yield up to 99.93%. Nanostructures of various geometries and materials can be reliably transferred onto diverse substrates that are rigid, soft, planar, and curved, even including a single-mode optical fiber. SENTL enables the fabrication of unconventional devices and the exploration of new applications. We demonstrate a compact spectrometer application using a convex lens integrated with diffractive nanogratings and SERS analysis for 2D materials through SENTL.

Electrostatic adhesion enables SENTL to achieve a high transfer yield; however, it also limits the transfer area because the large-area PVA thin film tends to be self-twisted due to the surface charges. The embedded PVA may also limit the resolution due to the capillary force in water immersion when the nanostructure spacing is too close. It is anticipated that SENTL may have exciting implications for electronic, plasmonic, and other types of devices that can benefit from its reliable responsiveness and broad compatibility.

## Materials and methods

### Electrodeposition process

Various metallic nanostructures can be grown through electrodeposition on ITO substrates. A two-electrode electrodeposition system (Keithley 2400 SourceMeter) was set up with ITO glass as the anode and 5 × 10 cm^2^ platinum-coated titanium grids as the cathode. The electrodeposition current densities were modulated and fixed at 5 mA/cm^2^ (Cu, Co) or 2.5 mA/cm^2^ (Au, Pd). Afterward, the deposited samples were thoroughly rinsed with deionized (DI) water, immersed in acetone to remove the resist, and then dried by nitrogen flow, leaving the electrodeposited metallic nanostructures on the ITO glass.

### SENTL process

The transfer carrier was applied on the patterned ITO substrate by spin casting 8 wt% PVA (Shang Hai Yingjia Industrial Development Co., Ltd, 97% hydrolyzed) aqueous solution at 500 rpm for 60 s and baking at 80 °C for 60 s. After curing, PVA was formed as a 15-µm-thick film (Fig. S1), of which the thickness was optimized to improve the transfer quality because it is difficult to achieve full-contact between thicker films and receivers, and thinner films are easily torn during subsequent separation. Next, the PVA carrier encapsulating the metallic nanostructures was slowly peeled off from the ITO donor using tweezers. Then, the flexible and thin PVA film was attached onto different receivers with a slight and uniform pressure of approximately 0.05 MPa on the backside. Subsequently, the receiver was heated at 70 °C for 20 s to purge the residual air in the PVA/receiver interface, which also further promoted adhesion. After that, the receiver was immersed in DI water for at least 60 min to dissolve PVA and then dried by nitrogen flow, leaving the nanostructures firmly attached.

### Morphology characterization

SEM (Zeiss Sigma 300 and Hitachi 4800) was performed to characterize the morphologies of the nanostructures. SEM images were analyzed by the commercial software ProSEM (GenISys Ltd.) to statistically calculate the feature size and roughness.

### KPFM measurements

KPFM was carried out using an atomic force microscopy (AFM) system (Multimode 8, Bruker). The surface potential was measured with one conductive probe (PFQNE-AL) on the same sample under two conditions. One measurement was done immediately after PVA film separation, and the other after releasing the triboelectric charges by repeatedly contacting the film with a clean Si wafer, and then placing the film on a stainless optical table for 24 h.

### Convex grating spectrometer characterization

The grayscale image was captured using a commercial monochromatic CMOS image sensor (QHY183M, Agena Astroproducts). The calibration of the wavelength and pixel number was achieved through quadric polynomial fitting. The reference spectrum was measured by a fiber optic spectrometer (USB 2000+, Ocean Optics).

### SERS analysis of graphene

The Raman scattering signal was collected for 8 s using confocal Raman microspectroscopy (LabRAM HR Evolution, HORIBA Jobin Yvon) with 785-nm excitation.

## Supplementary information

SENTL_Supporting Information_Finalized
