# Supplementary material for: High-fidelity and clean nanotransfer lithography using structure-embedded and electrostatic-adhesive carriers
Source: Microsyst Nanoeng. 2023 Jan 9;9:8. doi: 10.1038/s41378-022-00476-x (PMC9829746; doi:10.1038/s41378-022-00476-x)
Supplement: Supplementary file 1 — SENTL_Supporting Information_Finalized [file 41378_2022_476_MOESM1_ESM.docx]

Supporting Information

High-fidelity and Clean Nanotransfer Lithography Using Structure-embedded and Electrostatic-adhesive Carrier

Zhuofei Gan, Jingxuan Cai, Zhao Sun, Liyang Chen, Chuying Sun, Junyi Yu, Zeyu Liang, Siyi Min, Fei Han, Yu Liu, Xing Cheng, Shuhui Yu, Dehu Cui, and Wen-Di Li*

^∗^ E-mail addresses: liwd@hku.hk


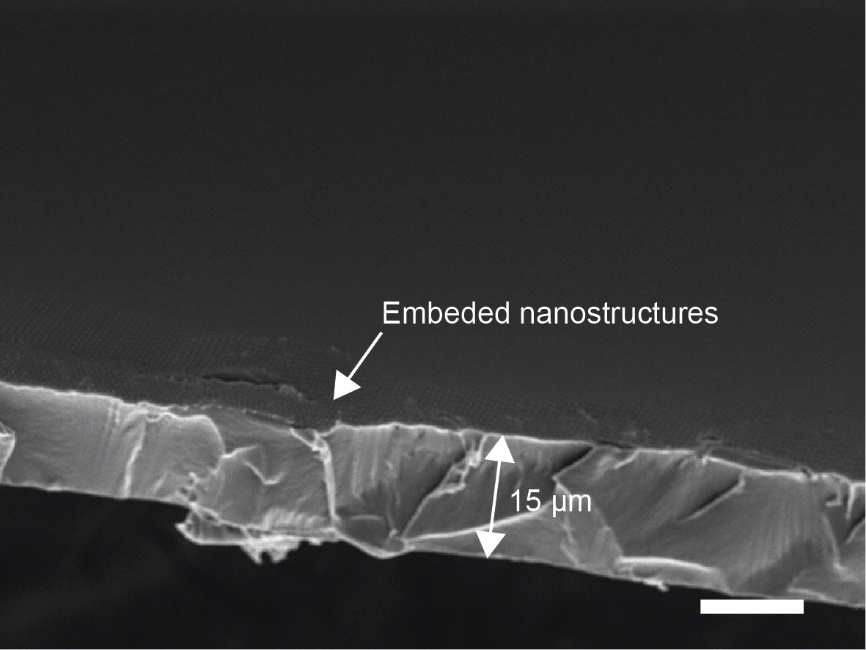


**Figure S1.** SEM image of the PVA film with nanostructures embedded. Scale bar, 10 μm.


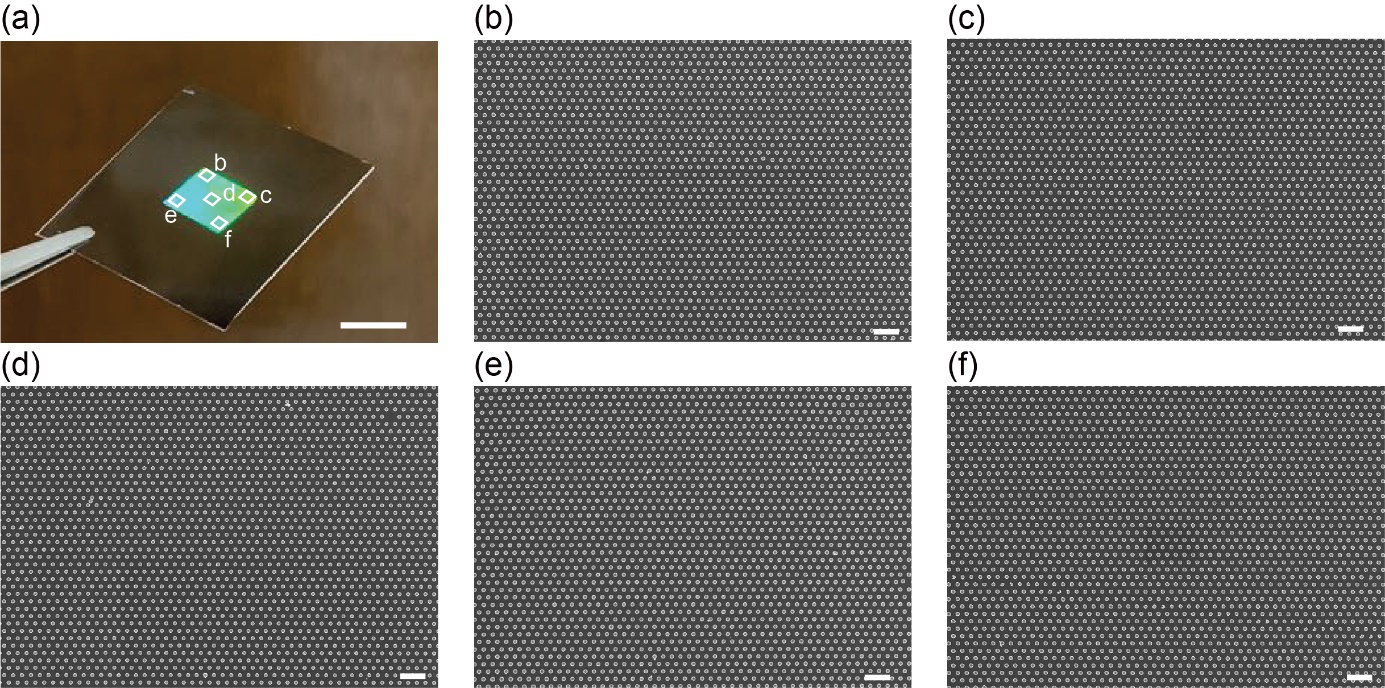


**Figure S2.** (a) Photograph of the 1-cm^2^-area transferred nanodisk pattern. (b-f) SEM images obtained from five different points on the transferred pattern. Scale bars, 2 μm in (b-f) and 1 cm in (a).


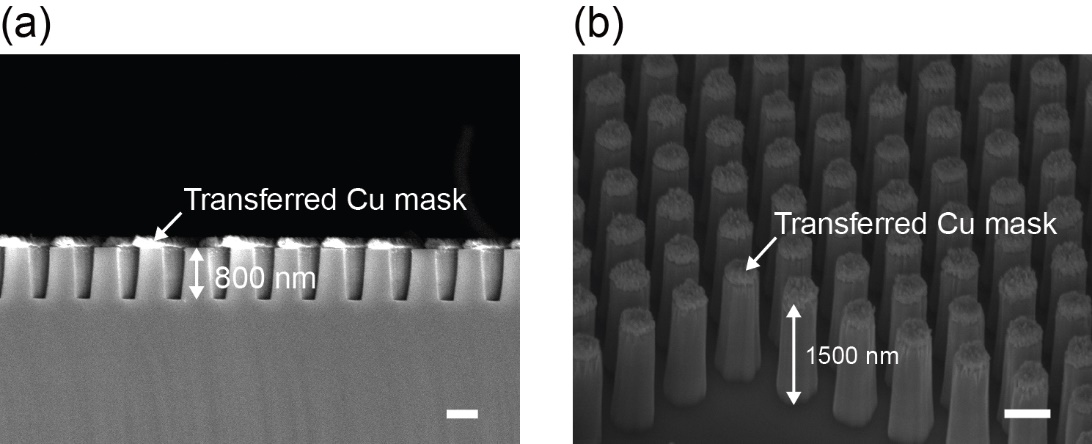


**Figure S3.** SEM images of the etched high-aspect-ratio Si nanostructures using transferred Cu pattern as the mask, (a) 800-nm-height nanogratings, (b) 1500-nm-height nanopillars. Scale bars, 500 nm.


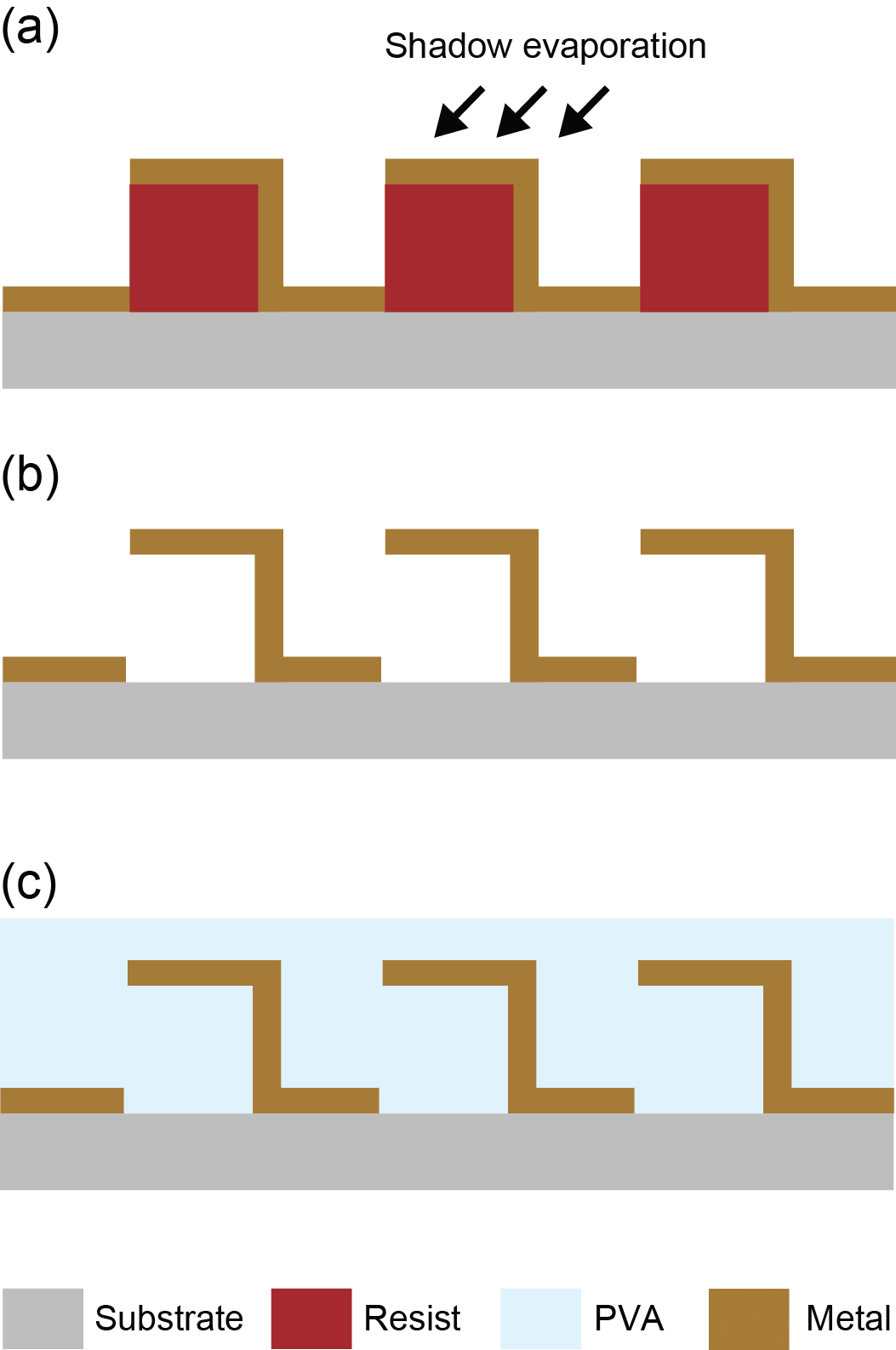


**Figure S4.** The schematic process flow of fabricating Z-shaped nanostructures. (a) Shadow evaporation on the resist grating. (b) Dissolving the resist. (c) Spin-casting PVA solution to fill the space among nanostructures.


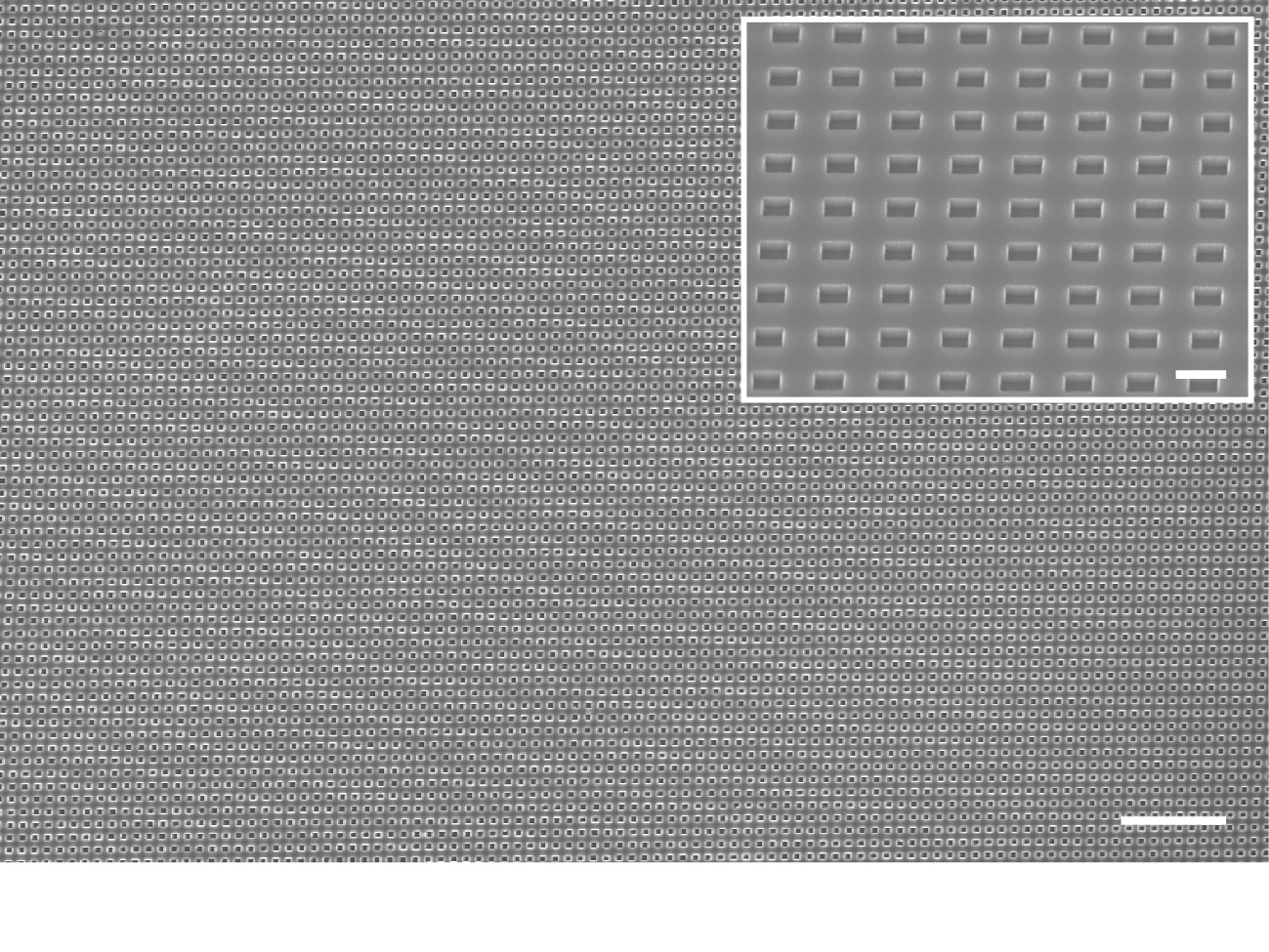


**Figure S5.** SEM images of the etched Si square nanohole array using transferred stack-mesh as the mask. Scale bars, 5 μm and 500 nm (inset).


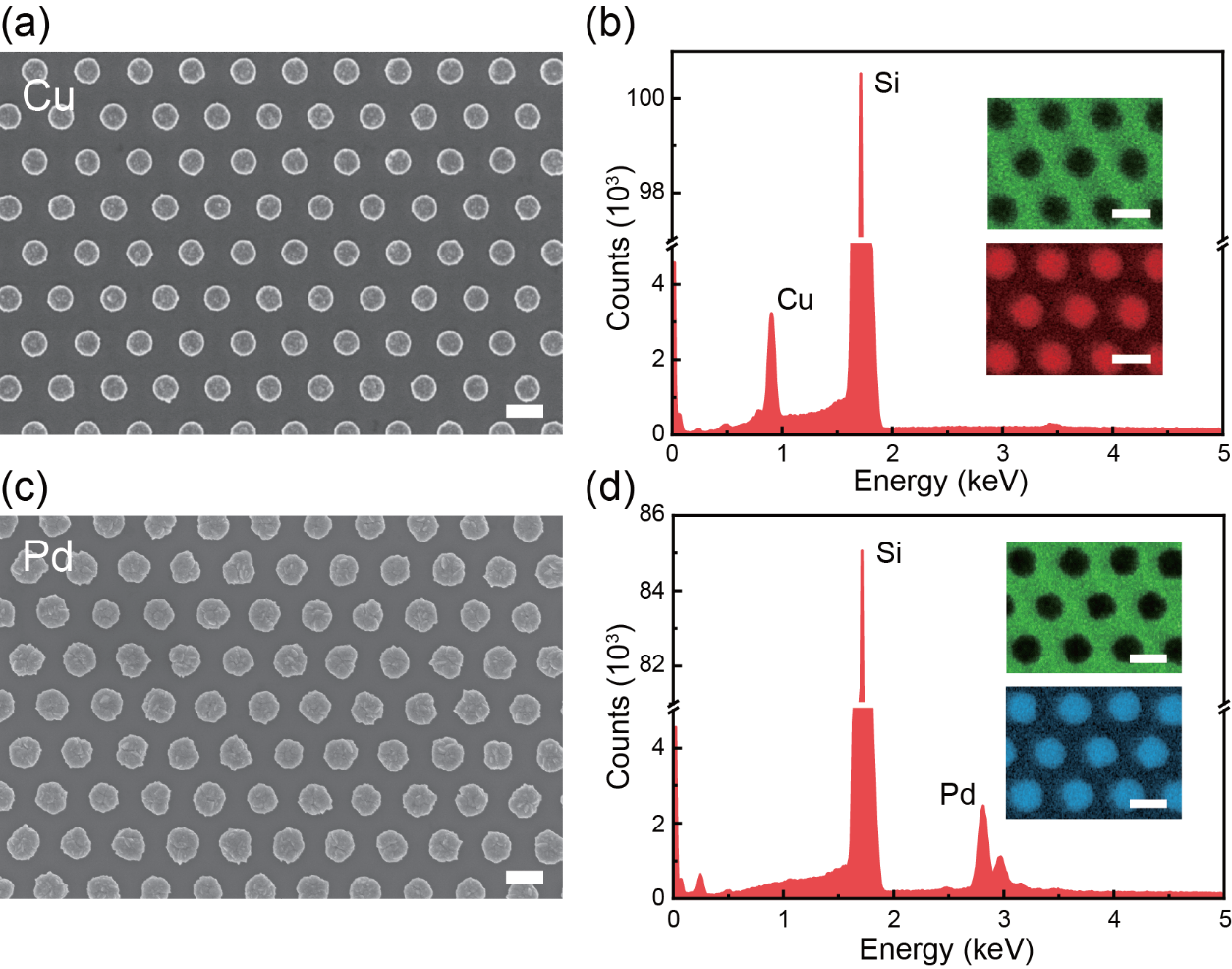


**Figure S6.** SEM images and corresponding EDX spectra and mapping of the transferred (a, b) Cu and (c, d) Pd nanodisks on Si. Scale bars, 500 nm.


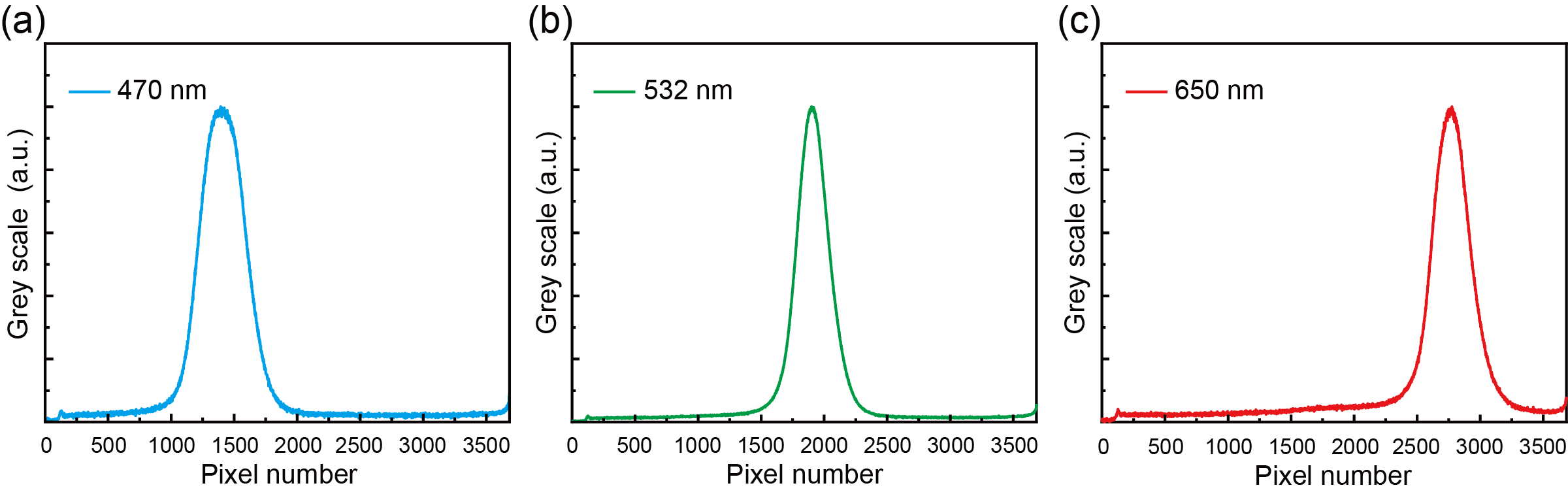


**Figure S7.** Spectra for wavelength calibration of (a) 470 nm, (b) 532 nm and (c) 650 nm.
